# 3D culture applied to reproduction in females: possibilities and perspectives

**DOI:** 10.1590/1984-3143-AR2023-0039

**Published:** 2024-03-08

**Authors:** Giuliana de Avila Ferronato, Franciele Flores Vit, Juliano Coelho da Silveira

**Affiliations:** 1 Faculdade de Zootecnia e Engenharia de Alimentos, Universidade de São Paulo, Pirassununga, SP, Brasil

**Keywords:** *In vitro* embryo, follicles, mechanotransduction, 3D culture system, tissue tension

## Abstract

*In vitro* cell culture is a well-established technique present in numerous laboratories in diverse areas. In reproduction, gametes, embryos, and reproductive tissues, such as the ovary and endometrium, can be cultured. These cultures are essential for embryo development studies, understanding signaling pathways, developing drugs for reproductive diseases, and in vitro embryo production (IVP). Although many culture systems are successful, they still have limitations to overcome. Three-dimensional (3D) culture systems can be close to physiological conditions, allowing greater interaction between cells and cells with the surrounding environment, maintenance of the cells' natural morphology, and expression of genes and proteins such as *in vivo*. Additionally, three-dimensional culture systems can stimulated extracellular matrix generating responses due to the mechanical force produced. Different techniques can be used to perform 3D culture systems, such as hydrogel matrix, hanging drop, low attachment surface, scaffold, levitation, liquid marble, and 3D printing. These systems demonstrate satisfactory results in follicle culture, allowing the culture from the pre-antral to antral phase, maintaining the follicular morphology, and increasing the development rates of embryos. Here, we review some of the different techniques of 3D culture systems and their applications to the culture of follicles and embryos, bringing new possibilities to the future of assisted reproduction.

## Introduction

Cell culture is a foundational procedure routinely conducted in many laboratories. The main objective is to maintain the cells in controlled conditions. (Ravi et al., 2015). This procedure has several applications, including in what is associated with assisted reproduction techniques, whether in basic embryo development studies to understand pathways or mechanisms, or in commercial *in vitro* embryo production (IVP). Although the monolayer (2 dimensional; 2D) system is the most used in cell culture, it still have limitations. In the 2D culture, cells lose their original shape and interact with the culture medium on just one side due to being attached to the plate dish. This can influence cell proliferation and differentiation (Jensen and Teng, 2020). Based on that, three-dimensional culture systems have emerged as an alternative to improve *in vitro* culture techniques (Gu et al., 2017).

**Figure 1:**
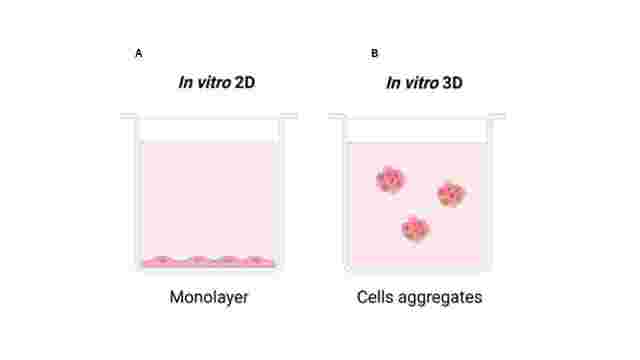
Representation of 2D and 3D cell culture systems. A) The 2D culture system led to a planar morphology covering the x-y plane and has a reduced height in the z plane. (B) in a 3D culture system, cells are allowed to interact with the media and other cells in all different positions around its membrane. Image created using Biorender (2023).

A 3D culture system is based on creating a cell microenvironment suitable and similar to *in vivo* conditions, which allows cells to explore its three dimensions, generating an increase in their interaction with the environment (Figure 1). Therefore, the 3D culture system presents advantages over 2D culture, as it allows the natural cell shape (Langhans, 2018), cell-to-cell communication (Souza et al., 2010; Ahn et al., 2020), gene expression as well as mechanical stimuli (Kaarj and Yoon, 2019), since 3D culture stimulates the extracellular matrix and induces cells to respond biologically to these physical signals (Ravi et al., 2015).

The first description of 3D culture was in 1912, using a surface made of silk threads for cardiomyocyte culture (Carrel, 1912). This culture system allowed greater interaction between cells and the surrounding environment. During the early 40s, Holtfreter (1944) performed different studies in developmental morphology describing a method to generate spherical cell aggregates by applying agar to the surface of Petri dishes, thus preventing cells from adhering to the bottom of the dish. In 1951, Joseph Leighton realized that 3D culture allowed cells to acquire their natural shape *in vitro*, thus presenting an increase in cell surface distinct from the monolayer cultures (Leighton, 1951). Due to Leighton’s contributions, he can be called the father of 3D cell and tissue culture (Hoffman, 2018).

The advantages of 3D culture systems attracted laboratories involved in several areas, such as, in new drugs development (Burgdorf et al., 2019, Tosca et al., 2023), since it can be an alternative to reduce animal experimentation, as well as in stem cell research since it allows greater aeration and nutrients (Wang et al., 2018). Besides that, studies are using 3D culture systems in experiments associated with reproduction, most of them related to early folliculogenesis (Pangas et al., 2003; Xu et al., 2011; Brito et al., 2014), and embryo culture (Zhao et al., 2015; Ferraz et al., 2018).

The 3D culture has come as an alternative to conventional cell culture, and it has been widely used in the most diverse areas of biomedical research. In this review, we provided an overview of 3D culture systems applied to animal reproduction as *in vitro* follicle culture and oocyte culture, in addition, to the application of different 3D cell culture techniques, and new perspectives of 3D culture applied for reproduction.

## Mechanical stimuli and reproductive tissue stiffness

Mechanotransduction is the cell’s ability to respond to mechanic stimuli, allowing cells to transform external physical stimuli into biological responses inside the cytoplasm, inducing pathways activation and modulating gene transcription (Martino et al., 2018). Thus, depending on the type of tissue, the extracellular environment can be soft or stiff, influencing cells' response (Discher et al., 2005).

**Figure 2:**
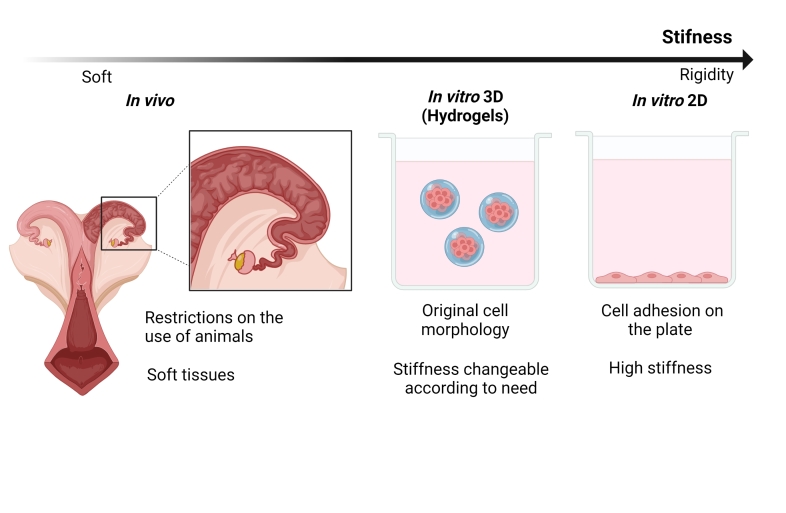
*In vivo* tissue characteristics compared to 3D and conventional *in vitro* cultures. Some materials used in 3D culture systems have stiffness like that found in the original tissue, creating a culture environment similar to what occurs *in vivo*. Adapted from Kolahi et al. (2012).

The reproductive tract presents tissues with soft tensions as the oviduct and the uterine epithelium which demonstrated stiffness between 100-1000 Pa (Kolahi et al., 2012). These mechanical stimuli are important for gametes and embryos, as these tensions form physical forces according to the needs of these cells (Figure 2). Conventional IVP or gametes culture, occurs on petri dishes, which have a stiffness of 1 GPa, six orders of magnitude (~10^6^) than that found in the uterine epithelium and oviduct. Some 3D culture systems, on the other hand, try to get around this through the use of different types of matrix, as is the case of materials based on hydrogels, which have a natural elasticity similar to the reproductive tract (around 1000 Pa) (Kolahi et al., 2012).

In mouse embryos, a higher blastocyst rate and a greater number of cells were obtained when *in vitro*-produced embryos were stimulated by continuous and uniform mechanical stimuli; mimicking what occurs naturally within the oviduct (Matsuura et al., 2010). Corroborating with that, mouse embryos produced in a soft matrix presented higher cleavage, blastocyst, and hatching rates as well as an increased number of total cells when compared with stiffer matrices (Kolahi et al., 2012).

The uterine tissue stiffness is involved in the establishment of pregnancy by embryo adhesion as well as in the adaptation of the organ as the fetus enlarges (Jorge et al., 2014). In the ovarian tissue, stiffness plays a role in follicle development impacting cell proliferation, differentiation, and growth (Jorge et al., 2014). A microfluidic system to culture secondary mouse follicles was generated using both alginate and collagen due to their differences in rigidity, mimicking the cortical part of the ovary with alginate (harder) and the medullary part with collagen (softer), as a result, they observed ovulation *in vitro*, in contrast to the conventional 2D culture, where they did not observe ovulation (Choi et al., 2014). This result emphasizes the relevance of mechanotransduction in biological processes and demonstrates how some 3D culture techniques can mimic *in vitro* the mechanical force originally found *in vivo*.

## 3D cultures techniques

**Figure 3:**
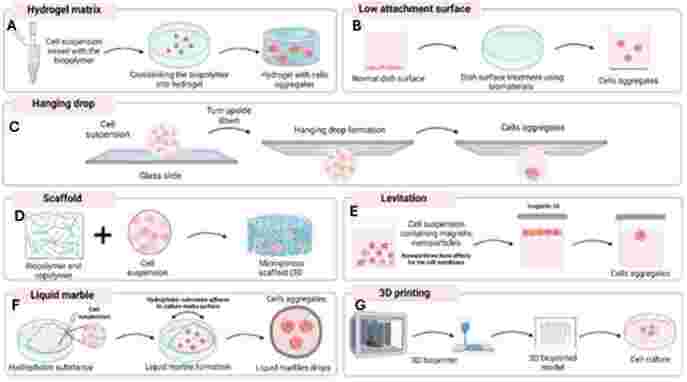
**S**chematic diagram of the different types of 3D culture techniques. (A) Using of hydrogel matrix to provide cell-to-cell interaction and thus created a 3D culture system. (B) Low attachment surface, the surface of the plate undergoes a modification (physical or chemical), causing the cells to form 3D structures. (C) Hanging drop consist of hanging droplets of cell suspension from the underside of a culture plate lid, which allows the cells to aggregate and form spheroids. (D) Scaffolds are three-dimensional (3D) structures that provide physical support and structure for the cells, they can mimic the native extracellular matrix, (E) Magnetic levitation, where magnetic nanoparticles are added to the cell culture medium, and a magnetic field is applied to levitate the cells. (F) Liquid marbles consist of a droplet of liquid (usually water) that is coated with a hydrophobic material (such as a surfactant or a polymer) to form a shell. (G) 3D printing/3D bioprinting is a technique that uses a (mix of polymers and biopolymers) to create 3D structures that mimic tissue environment. Image created using Biorender (2023).

Three-dimensional culture models can be applied to study several animals and organotypic explant cultures (including embryos), cell spheroids, microcarrier cultures, and tissue-engineered models (Haycock, 2011). Here we presented some different techniques used to create a 3D microenvironment (Figure 3).

### Hydrogels matrix

Hydrogels have been the subject of many studies in the follicle and embryo culture in different domestic species (Kolahi et al., 2012; Zhao et al., 2015; Vanacker and Amorim, 2017; Kim et al., 2020). Hydrogels are structures formed by water-soluble polymers, which form reticulated three-dimensional networks and can respond to external stimuli inducing the substance to become gel or liquid state, such as temperatures, pH, osmotic pressure, among other physical characteristics (Determan et al., 2007). There are several sources of hydrogel matrices, which are polymers or even components already present in the living cells' extracellular matrix.

The most commonly used matrix found in 3D studies is alginate (Vanacker and Amorim, 2017; Jones and Shikanov, 2019; Correia et al., 2020; Fuertes-Recuero et al., 2023), which is a natural polymer obtained from different species of brown algae. The alginate hydrogel is formed by the addition of a divalent ion, such as calcium, forming a stable solution with properties similar to the extracellular matrix (Lee and Mooney, 2012). Alginate hydrogel is well known for its non-cytotoxic proprieties, cell morphology maintenance, and antioxidant characteristics increasing cell viability (Jalayeri et al., 2017).

While alginate hydrogels are widely used for 3D cell culture, they present certain limitations. One of the primary constraints is their lack of bioactivity and limited capacity to mimic the complexity of native extracellular matrices. Alginate lacks the presence of cell-binding motifs and signaling molecules found in native extracellular matrices, which are essential for cell adhesion, proliferation, and differentiation(Sahoo and Biswal, 2021). Addressing these limitations often requires modifications to the alginate, such as incorporating bioactive molecules or combining it with other biomaterials. Natural components from the biological matrix can be also added to alginate hydrogel to improve the 3D environment (Xu et al., 2013). Fibrin can be added to allow cellular proteases to degrade the matrix and create a dynamic mechanical environment throughout the culture, (Jones and Shikanov, 2019; Zhao et al., 2023). Collagen and laminin, which are naturally present in the uterine extracellular matrix, can be added because of their elasticity that mimics the uterine environment, allowing the construction of very soft gels similar to the uterine tissue (Lopez-Garcia et al., 2010).

The utilization of decellularized extracellular matrix (dECM) hydrogels in 3D cell culture has been emerging as a powerful approach to create biomimetic microenvironments for cells. These hydrogels are derived from natural tissues that have passed through a decellularization process to remove cellular components while preserving the biological compounds. dECM hydrogels offer a complex blend of structural proteins, growth factors, and signaling molecules that closely mimic the native tissue microenvironment (Yao et al., 2019) . Researchers have been investigating dECM hydrogels for various applications, including regenerative medicine and disease modeling, highlighting the efficacy of dECM hydrogels in supporting cell adhesion, proliferation, and differentiation, making them invaluable tools for advancing our understanding of tissue biology and facilitating tissue engineering strategies (Nishiguchi and Taguchi, 2021; Zhao et al., 2021; Kim et al., 2022).

### Hanging drop

The hanging drop (HD) method was initially developed for microbiology, for bacterial studies (Swift, 1963). However, HD reveals promising results when adapted for animal embryology studies and has been used extensively in embryoid bodies (Ohnuki and Kurosawa, 2013; Behringer et al., 2016; Wu et al., 2016).

The HD is a simple technique; it consists of using gravity to form spheroids from the aggregation of cells. The cells are suspended together with the culture medium in an inverted plate and are incubated, thus forming the spheroid (Eder and Eder, 2017). The HD can also be associated with other materials to improve the technique, such as methocel and matrigel (also called “reconstituted basement membrane”) because some cell lines need these conditions to form spheroids (Haeger et al., 2011).

HD has already been used in granulosa cell cultures from buffalos to mimic the intra-follicular environment (Yadav et al., 2018) and in porcine oocytes to investigate antioxidant factors for maturation (Ishikawa et al., 2014). Hang drop culture can be a simple and low-cost method to obtain 3D structures for cell study.

### Low attachment surface

The low attachment surface technique consists of modifying the surfaces of culture dishes to prevent cell adhesion, promoting cell-to-cell interactions (Su et al., 2013; Tsai et al., 2021). To study the immune tolerance and proper human embryo implantation, Alexandrova et al. (2022) constructed stable, reliable, and reproducible trophoblast Sw71 spheroids independently of the serum level in the culture media. These models are similar to hatched human blastocysts in size, shape, and function. The Sw71 cells were cultured in a U-bottom 96-well low attachment plate. After 8 h the Sw71 formed loose aggregates followed by compaction (12-24 h) and the formation of a single differentiated stable spheroid with an intact periphery.

Ren et al. (2023) used the low attachment surface technique to investigate an optimized 3D system for preantral follicle culture. The authors isolated oocytes from mouse ovaries, these ovaries were placed in ultra-low attachment 96-well plates and randomly divided into 2 groups. One group was supplemented with FBS or bovine serum albumin (BSA) and the other group was encapsulated with an alginate supplement with FBS or BSA culture. The follicular diameter was measured, and the lumen of the follicle was evaluated. Also, they tested the ability of these oocytes to be fertilized *in vitro*. As a result, the diameters were larger for the growing secondary follicles cultured in ultra-low attachment 96-well plates than in the alginate gel, however; the fertilization rate was not different. This study presented insights into the mature oocytes obtained from the 3D culture of the preantral follicle by using an ultra-low attachment 96-well plate with an FBS-free for *in vitro* fertilization. Thus, the use of surfaces with low adhesion can be a good alternative as it does not require matrices or other reagents to form 3D cellular structures.

### Scaffold

Nowadays, the interest in reproductive tissue engineering (REPROTEN) has increased since this application can improve the quality of life of patients with reproductive dysfunctions. In this case, to develop healthy tissue, the scaffold is the technique mostly used (Amorim, 2017). Scaffolds are 3D structures constructed by porous biomaterials, fibrous or permeable structures (Edmondson et al., 2014). These structures increase the transport of liquids and promote better cell-to-cell interaction. Scaffolds applied for reproduction studies have shown favorable results (Nikolova and Chavali, 2019).

The scaffold can be developed using different polymers as biopolymers such as gelatin, and collagen, and synthetic polymers as poly(epsilon-caprolactone) (PCL). Commonly, blends of biopolymer and synthetic polymer are used to provide a robust and biocompatible structure (Liverani et al., 2019). Di Berardino et al. 2022 designed poly(epsilon-caprolactone) (PCL)- based electrospun scaffolds with different topologies to compare ovine preantral follicles *in vitro* culture with a conventional culture. PCL scaffolds were able to support follicle growth, antrum formation, and the upregulation of follicle marker genes leading to a greater oocyte meiotic competence than in the 3D-oil system (Di Berardino et al., 2022)

Eissa et al. (2018) developed a scaffold using porous polymers (known as polyHIPEs) for the culture of primary human endometrial epithelial and stromal cells (HEECs and HESCs). The infiltration of HEECs and HESCs into cell-seeded polyHIPE scaffolds was assessed by histological studies, and the phenotype was confirmed by immunostaining. The differentiation of HEECs and HESCs in polyHIPE scaffolds and monolayer cultures was evaluated by monitoring the expression of endometrial marker genes. The author observed that a 3D cell culture formed an endometrial with architecture and function like *in vivo* endometrial when it was compared to a 2D cell culture.

The use of natural scaffold has grown in the recent years. Decellularized ECM-based scaffolds may offer a new promise for the reconstruction of ovaries of patients that suffering from infertility caused by cancer (Laronda et al., 2015; Laronda, 2020; Pennarossa et al., 2020a, 2021). The decellularization process of scaffolds needs to preserve the original architecture and mechanical properties of the scaffolds. To achieve the most effective protocol, the decellularization process involves a combination of physical (scraping, sonication, and agitation), chemical (detergents and alcohols), and biological (*e.g.* trypsin, nucleases, collagenase, lipase, and dispase) techniques (Crapo et al., 2011). Laronda et al. (2015) created a decellularized ovarian scaffold, recellularized and transplanted this scaffold in ovariectomized mice. It was observed that the transplanted ovarian scaffold was able to initiated puberty in the ovariectomized mice. These results may provide a new technique which can be used to drive future human ovary transplants.

### Magnetic levitation

Magnetic levitation culture systems are based on magnetic nanoparticles with a less than 1 μm size composed of magnetic elements such as iron, and nickel, among others. First, the magnetic elements are placed together with the cells that will be cultured, after a while these nanoparticles adhere to the cell membrane. Subsequently, these cells conjugated with magnetic beads are subjected to a magnetic field to induce cell aggregates by levitation, forming three-dimensional spheroids or ring-shaped structures (Turker et al., 2018).

This system allows a practical and efficient 3D culture. Antonino et al. (2019) used the levitation technique to observe the structure and viability of ovarian follicles. The author observed that ovarian follicles cultured using magnetic levitation showed higher viability, antrum formation, and lower degeneration rate. Thus, magnetic levitation can be a potential technique for follicle culture as an alternative to conventional systems.

### Liquid marble

Liquid marble (LM) is the result of an interaction between an extremely hydrophobic substance (external phase) and the culture medium containing the cells (internal phase) (Avrămescu et al., 2018). The hydrophobic substance adheres to the drop of liquid forming an enclosed microenvironment that induces a 3D system. This technique has many advantages, such as easy handling, the droplet size can be variable allowing multiple or individual cultures depending on the needs of each study, additionally, it allows the addition of drugs to be tested, the use of low volumes of culture media, and it has a very low-cost (Ledda et al., 2016). LM has multiple applications and has been used in sheep oocytes during *in vitro* maturation, using a total of 10 cumulus-oocyte complexes (COCs) per drop (Ledda et al., 2016; Bebbere et al., 2021). Thus, this type of system may improve de diffusion of the medium for COC *in vitro* maturation increasing the blastocysts rates. However, recently the LM system was used during oocyte maturation and embryo culture (Ferronato et al., 2023). The LM system induced a decrease in transcripts related to oocyte maturation process in cumulus cells (Ferronato et al., 2023). Importantly, the LM system applied during IVC induced a decrease in blastocyst rate and total cell number while an increase in global DNA methylation and hydromethylation levels (Ferronato et al., 2023). Although the results in bovine were not improved, the LM system demonstrated to induce epigenetic changes, which if combined with other changes in media and gas pressure could lead to better results.

The LM also can be used as a micro-bioreactors, to promote 3D cell rearrangement to extend and stably maintain high plasticity *in vitro* (Pennarossa et al., 2019, 2020a). Arcuri et al. (2024) and Pennarossa et al. (2023), generated inner cell mass (ICM)-like organoids using a LM micro-bioreactor to promote 3D cell rearrangement and boost pluripotency. The cells encapsulated into micro-bioreactors rearrange in 3D ICM-like structures, creating structures called epiBlastoids, which can be used as models for studying early embryo development (Arcuri et al., 2024; Pennarossa et al., 2023).

### 3D printing and 3D bioprinting

3D printing is a process that creates three-dimensional objects by adding material layer by layer. This technique allows to development of molds with high precision and accuracy, these molds can be used to create scaffolds or microdevices to study complex structures (Chen et al., 2011). The 3D printing mold may be associated with other types of 3D cultures as hydrogels (Laronda et al., 2017). Also, it can use biopolymers as the ink for 3D printing, achieving interesting molds for cell culture (Do et al., 2015). 3D printing can also be used to print devices for cell culture, such as microfluidic chips, for example. Ferraz et al. (2017) developed an oviduct-on-a-chip using 3D printing to create the design of a microfluidic chip. The microchip was able to mimic the bovine oviduct and was successful in allowing fertilization of the oocytes by the spermatozoa, as well as avoiding polyspermy.

Nowadays, the use of 3D bioprinting has increased. The main outcome is the development of a good mix of polymers and hydrogels to create an appropriate bioink for the cells. 3D bioprinting is very similar a 3D printing, but this technique can print the structure with cells in just one step. Nie et al. (2023) used 3D bioprinting to construct a bilayer human endometrial construct (EC) based on a sodium alginate-hyaluronic acid (Alg-HA) hydrogel for functional regeneration of the endometrium. They restored the morphology and structure of the endometrial wall (including organized luminal/ glandular epithelium, stroma, vasculature, and the smooth muscle layer), as a result they were able to improve the reproductive outcome in the surgical area after implantation (75%) (Nie et al., 2023).

Wu et al. (2022) developed a 3D bioprinting of artificial ovaries by an extrusion-based, using gelatinmethacryloyl (GelMA) as bioink. They used cells from the ovaries of 4-week-old female C57BL/6J mice and after passage two, the cells were trypsinized and prepared for cell-laden bioprinting. The GelMA-based 3D printing system provided an appropriate microenvironment for ovarian follicles, which successfully grew and ovulated in the scaffolds (Wu et al., 2022). 3D printing and 3D bioprinting are tools that allow the creation of experimental settings simulating the stiffness, physiology, and morphology of natural tissues.

## 3D culture applied to the female reproductive system

Most studies of 3D culture in the female reproductive system are focused on follicles and embryos, However, some research studies the culture 3D of the reproductive tract as oviduct and ovaries. Here, we presented some applications using the techniques presented above.

### 3D culture applied to ovarian follicle culture

Ovarian follicle culture is an important technique for understanding folliculogenesis, cryopreservation, and toxicology tests for fertility-related drugs, among others (Vanacker et al., 2012; Zhou et al., 2015; Rossetto et al., 2016). In 2D culture, the follicles lose their original architecture due to the adhesion of granulosa cells to the bottom of the plate, inducing changes in the cell-to-cell interactions and between somatic cells and the oocyte (Gutierrez et al., 2000; Picton and Gosden, 2000). Thus, several researchers are using 3D systems formed from different types of matrices (Table 1) to simulate the physiological ovarian environment to improve follicle culture (Vanacker and Amorim, 2017; Antonino et al., 2019; Jones and Shikanov, 2019; Shen et al., 2020).

**Table 1:** 3D culture systems and their main results in reproduction studies.

**3D cell culture technique**	**Material**	**Cells**	**Results**	**References**
Hydrogel	Alginate	Follicle	Greater viability and differentiation of pre antral into antral follicles in countless animal species	(Vanacker et al., 2012)
		Embryo	Greater embryo viability after hatching and embryo elongation	(Zhao et al., 2015)
		Spermatogonial stem cell	Keep cells viability and morphology	(Jalayeri et al., 2017)
	Extracellular matrix-derived	Follicle	Greater antrum formation, maturation rate, and normal spindle	(Kim et al., 2020)
	Collagen + Alginate	Follicle	Oocytes ovulation	(Choi et al., 2014)
	Collagen	Embryo	Higher cleavage, blastocyst, and hatching rates. A greater number of trophoblast cells	(Kolahi et al., 2012)
Magnetic Levitation	Magnetic nanoparticles	Follicle	High follicular growth, oocyte viability, morphology, and maturation rate.	(Antonino et al., 2019)
Hanging drop	-	Granulosa cell	Upregulate CYP19 expression, simulating preovulatory follicle stage	(Yadav et al., 2018)
Low attachment surface	Non-adherent surfaces	Oocytes	Growing secondary follicles’ diameters were larger when cultured in ultra-low attachment 96-well plates than in the alginate gel	(Ren et al., 2023)
Scaffold	Biopolymer and matrix	Primary human endometrial epithelial and stromal cells (HEECs and HESCs).	3D cell culture formed an endometrial architecture and function similar to *in vivo* endometrial than 2D cell culture	(Eissa et al., 2018)
3D printing	Polymer combination	Ovary/ endometrium	Ovarian bioprosthetic able to return fertility in sterile mice	(Laronda et al., 2017)
			improved the reproductive outcome in the surgical area after implantation (75%,)	
				(Nie et al., 2023)
3D biprinting	biopolymers combinations			

Alginate hydrogel has been the most used biomaterial tested for ovarian follicles 3D culture systems in domestic animals (Vanacker and Amorim, 2017), due to its promising results combined with easy manipulation, *in vivo* biocompatibility, and in vitro non-cytotoxicity (Eaton et al., 1990); Pangas et al., 2003; Lee and Mooney, 2012; Brito et al., 2014; Jalayeri et al., 2017). In non-human primates, Xu et al. (2011) demonstrated the ability of secondary follicles to grow in alginate hydrogels to reach oocyte maturation until metaphase II (MII), and using the right concentrations of fetuin, FSH, and 5% of O_2_ in the culture medium, they also obtained a greater survival of follicles and greater production of AMH after the antrum formation. In goats, the use of alginate hydrogel allowed follicle activation and continued growth of primordial follicles (Correia et al., 2020).

In cattle, secondary follicles were able to reach the antral phase in alginate hydrogel culture for 32 days; additionally, it was observed that the addition of growth hormone (GH) was able to increase the estradiol production (Araujo et al., 2014).

Kim et al. (2020) compared alginate hydrogel with extracellular matrix-derived soft hydrogel (ES-hydrogel). It was observed greater antrum formation, higher maturation rate, normal spindle morphology in oocytes as well as normal E2 production in ES-hydrogel. To achieve these results authors compared the lower rigidity of ES-hydrogel to alginate hydrogel, which in addition to maintaining the structure of the follicle also allowed a better exchange of nutrients and hormones with the medium.

Antonino et al. (2019) used magnetic levitation, using magnetic nanoparticles to culture secondary bovine follicles, and were able to obtain greater follicular growth, antrum formation, better morphology, oocyte viability, and higher oocyte resumption rate after *in vitro* maturation. Shen et al. (2020) cultured buffalo oocytes in 3D utilizing a glass scaffold system. Based on this method, Shen et al. (2020) obtained higher oocyte maturation, cleavage, and blastocyst rates as well as greater blastocyst cell numbers. They also found higher levels of proteins related to oocyte maturation (COL1A1, COL2A1, COL3A1, and FN) in cumulus cells as well as cell connection-related proteins such as N-cadherin, E-cadherin, and gap junction alpha-1 protein (GJA1), indicating that 3D culture might promote oocyte maturation due to improvement in cell-to-cell connection (Shen et al., 2020).

To optimize *in vitro* maturation for ovine was development a three-dimensional (3D) scaffold-mediated follicle-enclosed oocytes with ovarian surface epithelium cells. This system was compared with a conventional cumulus-oocyte complex (COC) protocol. The 3D scaffold promoted synergic cytoplasmatic and nuclear maturation, offering a novel culture strategy to widen the availability of mature gametes for ART (Peserico et al., 2023).

A crucial point around ovarian follicles is the preservation of fertility in females, which seeks to maintain the oocyte viability for future use. Hydrogels are considered good encapsulation material to protect cells during cryopreservation, as they create barriers preventing the formation of harmful ice crystals (Bhakta et al., 2009). Some studies illustrate success upon vitrification of follicles with alginate capsules (Vanacker et al., 2012; Bian et al., 2013), although others studies still demonstrate low viability of these follicles compared with fresh follicles (Sadeghnia et al., 2016; Bus et al., 2018; Sadr et al., 2018), demonstrating the need to improve follicle vitrification protocols, 3D culture for follicle present as a good strategic since 2D cannot mimetic the cell-cell interaction that occurs inside the follicles.

### 3D culture applied to embryo culture

*In vivo,* embryos in the early stage of development are allocated in the oviduct, which is an extremely important organ for embryo development (Li and Winuthayanon, 2017) In vitro, embryos are normally produced in 2D culture systems, using culture dishes. Although IVP is a well-established technique, some limitations need improvements, such as lower blastocyst and birth rates (Jaguszeski et al., 2019), and decreased resistance to cryopreservation (Sanches et al., 2017) compared with *in vivo*-produced embryos.

In addition, embryos produced *in vitro* have epigenetic changes when compared to embryos produced *in vivo* (Canovas et al., 2017). These changes are regulated by DNA methylation, post-translational modifications of histones, and microRNAs (miRNA) that can influence embryo development or even its health after birth (Bouillon et al., 2016).

To overcome the 2D system limitations, 3D culture systems could be a potential alternative since it can mimic a physiological environment. Kolahi et al. (2012) demonstrated that mouse embryos cultured in a 3D system composed of type I collagen, presented higher cleavage, blastocyst, and hatching rates as well as an increased number of trophoblast cells compared to conventional *in vitro* embryo culture. This can be explained by the mechanical properties of the environment, since collagen is already part of the extracellular matrix of the uterus, thus its use can give a natural elasticity to the *in vitro* culture, similar to the uterine environment (1 kPa) (Discher et al., 2005; Filas et al., 2011).

Interestingly, embryos produced in 3D and conventional culture systems generate the same number of fetuses after transfer to recipient cows; however, the weight of the placenta was greater in the 3D group than in the conventional culture group. This result demonstrates that in addition to affecting the initial embryonic development by the difference in the number of trophoblast cells, the environment in which the embryo is inserted can also affect embryos after implantation, due to the difference in placental weights (Kolahi et al., 2012).

Corroborating with this, some studies also associate the beneficial effects of 3D systems with the mechanical pressure naturally exerted by the zona pellucida (ZP). In mice, a study removed the ZP of embryos before culture in an alginate matrix combined with calcium and was able to obtain better blastocyst rates in comparison to ZP-free embryos cultured without matrix (Eaton et al., 1990).

In bovine, Zhao et al. (2015) used alginate as a method to maintain embryo architecture after hatching to build a system for embryo elongation and implantation studies, since it is hard to maintain embryos' normal morphology *in vitro*, due to cell adhesion to the culture plate and interruption of cell-to-cell interactions. The embryos were cultured for 18 days and those that were encapsulated with alginate had a higher survival rate and were able to present expansion and elongation after 18 days (Zhao et al., 2015). Once embryos were placed back in conventional culture, they still showed growth, until the 26th and 32nd day of culture as well as demonstrated the presence of binuclear cells and expression of genes associated with placental tissue differentiation (Zhao et al., 2015).

Due to the lack of knowledge in early embryonic development, creating studing models that mimic embryos in this stage are necessary, with this, blastocyst-like structures were developed. The morphology and cell lineages of animal stem cell-derived blastoids are comparable to those of normal blastocysts in recent research (Knospel et al., 2019). Li et al developed an entire blastocyst using a single stem cell type from a mouse blastomere (8 cells). The authors established a 3D differentiation system that enabled the generation of blastocyst-like structures. Embryonic pluripotent stem cells derived blastoids present a similar structure to a blastocyst in terms of morphology and cell-lineage allocation (Li et al., 2019).

These results are promising for the study of *in vitro* fertilization and embryo development which can help us to understand what occurs physiologically, thus improving existing IVP techniques as well as development rates of *in vitro* produced embryos.

### 3D culture applied to female reproductive tract models

In addition to ovarian follicles and embryos, 3D culture systems are also applied to reproductive tract models, to understand biological pathways or transplants for fertility recovery. MacKintosh et al. (2015) using a 3D model of the bovine primary endometrium culture, demonstrated that epithelial and stromal cells can be cultured in an electrospun polyglycolide (PGA) scaffold, resembling *in vivo* cell organization. This achievement allowed the investigation of pathophysiological diseases and the development of new therapies (MacKintosh et al., 2015).

In the early stages of embryonic development, the yolk sac (YS) performs a crucial role in performing hematopoietic, metabolic, and nutritional functions. In this context, Pereira et al. (2023), developed a three-dimensional (3D) culture model of the YS of three different domestic species: canine, bovine, and porcine. The authors observed that 3D YS models demonstrated improved cell organization and morphological similarity to the original tissue compared to the standard 2D model (Pereira et al., 2023).

3D Organoids can be used to investigate the normal biology and pathology of the female reproductive tract (FRT). The FRT includes the ovaries, fallopian tubes, endometrium, and cervix, as well as placental trophoblast (Alzamil et al., 2021). Kopper et al develop a 3D organoids platform to study ovary cancer (OC) in vitro. They selected 56 organoid lines from 32 patients' cultures in cold Cultrex growth factor. The lines represent all main subtypes of OC. They used these OC organoids for drug-screening assays and observed chemoresistance. The organoids present a long-term expansion and can be genetically modified. In addition, these 3D organoids also can be xenografted, enabling *in vivo* drug-sensitivity assays. This platform can be used as a potential application for personalized medicine (Kopper et al., 2019).

Studies using 3D organoid cultures from human fallopian tubes can be powerful in understanding the origin of high-grade serous ovarian cancer. Kessler et al. (2015) establishment of long-term, stable 3D organoid cultures from human fallopian tubes. In this research, was observed that single epithelial stem cells in vitro can give rise to differentiated organoids containing ciliated and secretory cells. The authors performed a microarray analysis and observed that inhibition of Notch signaling causing downregulation of stem cell-associated genes in addition to decreased proliferation and increased numbers of ciliated cells (Kessler et al., 2015).

Ferraz et al. (2018) built a device using 3D printing to mimic the oviduct. They showed that the DNA global methylation of zygotes produced in the “oviduct-on-a-chip” system were similar to embryos produced *in vivo*, and differed from embryos produced in vitro, as well as the level of transcription of genes linked to DNA (de)methylation.

On the other hand, Xiao et al 2016 developed a microchip to study a human 28-day menstrual cycle hormone profile. They used a murine ovarian follicle and placed it inside of microchip. Due to a dynamic flow rate it was possible to simulate the *in vivo* female reproductive tract and the endocrine loops between organ modules including the ovary, fallopian tube, uterus, cervix, and liver. The organ-organ platform presented a great potential to be used in drug discovery and toxicology studies (Xiao et al., 2017)

In mice, a study was able to perform a bioprosthetic ovary using 3D printing together with hydrogel matrices (Laronda et al., 2017). Follicles in the ovary developed normally, were able to ovulate, and the oocytes were fertilized leading to healthy and fertile offspring (Laronda et al., 2017). These studies demonstrate the versatility of 3D culture systems, suggesting their use in several different approaches to contribute to basic and applied science.

## Challenges and limitations observed in 3D culture systems

Although 3D cultures are most accurate in physiological terms, 3D culture cells can present challenges as standardizing a culture protocol and designing experimental studies of drug delivery and lytic assays (Jensen and Teng, 2020; Habanjar et al., 2021).

Moreover, the addition of more components (*i.e.;* matrices) increases the complexity of the system and makes the incorporation of medium and drug more difficult due to the increase in the volume of medium and drugs (Montanez-Sauri et al., 2013).

Furthermore, to verify the cellular response in 3D cultures it is common to use an immunocytochemistry (ICC) assay. Although the ICC assay can provide information regarding the localization, concentration, and activation of biomolecules, the quantification of fluorescent signals is often interrupted by background signals and the non-specificity of primary or secondary antibodies. Therefore, conventional biochemical assays (*i.e*., ELISA, western blots, qPCR) need to accompany the ICC assay analysis (Berry et al., 2011; Montanez-Sauri et al., 2015). However, 3D culture presents advantages when compared to its analog (2D culture). The 3D culture provides an environment close to *in vivo* system, allowing one to understand reliably, the embryo development process (Goodman et al., 2008; Knight and Przyborski, 2015; Ravi et al., 2015)

## Future perspectives

3D culture is presented as a good alternative to mimic a physiological system. In this review, we described some strategies to develop microenvironments that allow to observe the behavior of 3D structures when compared with 2D culture (conventional culture).

The 3D culture allows the use of different hydrogels (combined or isolated) to develop an appropriate environment. Also, several techniques can be applied as 3D printing and bioprinting, scaffolds, magnetic levitation hang drops, and liquid marbles to create robust structures. (Xu et al., 2011).

3D culture has demonstrated relevant results for new drug development (Jensen and Teng, 2020). In embryology the number of studies using 3D culture for the embryo, follicles, and female environment has increased In the last years. However, 3D still presents lack of validation, especially in early embryo development, where studies are scarce and need additional tests with different types of matrices and as well as in different species. Therefore, studies focused on understanding the different mechanisms using 3D culture may be beneficial in ART, since 3D culture presents some of the results similar to the obtained for the *in vivo* cells.
